# Diagnosis and Treatment of Uveitis in Children: A Summary of the Latest Data from a 5-Year Literature Review (2018–2023)

**DOI:** 10.3390/jcm13113097

**Published:** 2024-05-25

**Authors:** Monika Modrzejewska, Oliwia Zdanowska

**Affiliations:** 1 Second Chair and Department of Ophthalmology, Pomeranian Medical University in Szczecin in Poland, Al. Powstańców Wlkp. 72, 70-111 Szczecin, Poland; 2 K. Marcinkowski University Hospital in Zielona Góra, 65-046 Zielona Góra, Poland

**Keywords:** pediatric uveitis, diagnosis, treatment, review, optical coherence tomography

## Abstract

Pediatric uveitis has a low incidence. It is very diverse in its presentation and is often the first sign of a severe systemic disease. The pediatric population poses a special therapeutic and diagnostic challenge due to the potentially adverse effects of therapeutic agents on the young body and difficult cooperation with the patient during the examination, as well as the increased risk of complications that can lead to severe disability. The most commonly diagnosed type of uveitis is non-infectious, with first-line therapy consisting of systemic corticosteroids followed by disease-modifying drugs (methotrexate (MTX), mycophenolate mofetil (MMF), and cyclosporin A (CsA)). In severe, refractory cases, biologic therapy is used. The authors reviewed the current literature on the etiology, diagnostic tools, and treatment of uveitis in the pediatric population covering the years 2018–2023, presenting current methods of modern diagnosis and treatment. The reason for writing this article was the need to update the knowledge on uveitis, driven by the increasing prevalence of autoimmune uveitis in the pediatric population. This trend presents significant challenges in diagnosing and treating the disease, as well as managing its complications. Correctly identifying the pathogenetic factor of uveitis can facilitate the diagnosis of the systemic disease underlying the ocular infection and enable the timely implementation of systemic treatment. Furthermore, the emergence of new diagnostic methods necessitates a revision and update of ophthalmic knowledge, essential for both ophthalmologists and other specialists involved in the treatment of uveitis.

## 1. Introduction

Juvenile uveitis is an inflammatory disorder involving intraocular structures, including the choroid, iris, and ciliary body. It occurs in the course of a variety of severe systemic diseases. The current classification of uveitis is based on the criteria of the Standardization of Uveitis Nomenclature Working Group (SUN) of 2021, where a classification was established for the 25 most common types of uveitis [1]. Regarding pathogenesis, the classification of uveitis includes infectious, non-infectious, and idiopathic factors. Considering the localization of inflammation, uveitis is divided into anterior uveitis, intermediate uveitis, posterior uveitis, and panuveitis. The incidence of uveitis in the pediatric population accounts for about 5–10% of all cases of uveitis [2]. Children with uveitis present a diagnostic and therapeutic challenge for ophthalmologists. Pediatric patients have a limited ability to verbalize their symptoms and to cooperate during examinations. A factor that worsens the prognosis in young patients is the increased risk of ocular complications due to ongoing inflammation, which may include glaucoma, cataracts, band keratopathy, and inflammation of the vitreous body, retinal vessels, and/or the optic nerve [3]. Uveitis in the pediatric population is often a manifestation of severe systemic diseases, necessitating multispecialty care and increasing the risk of complications in other organs. The adverse effects of therapeutic agents on a child’s development pose challenges in selecting appropriate therapy. Appropriate diagnosis, based on specialized imaging techniques of intraocular structures, multispecialty cooperation, and a carefully chosen therapeutic regimen, is crucial in the care of children with uveitis. Due to rapid medical advancements, there is much literature on the diagnosis and treatment of uveitis, including newer methods that are extensively described in relation to the adult population but much less frequently in relation to children.

The following literature review aims to present the most up-to-date reports on the diagnosis and therapy of uveitis in the post-pediatric population.

## 2. Materials and Methods

**Figure 1:**
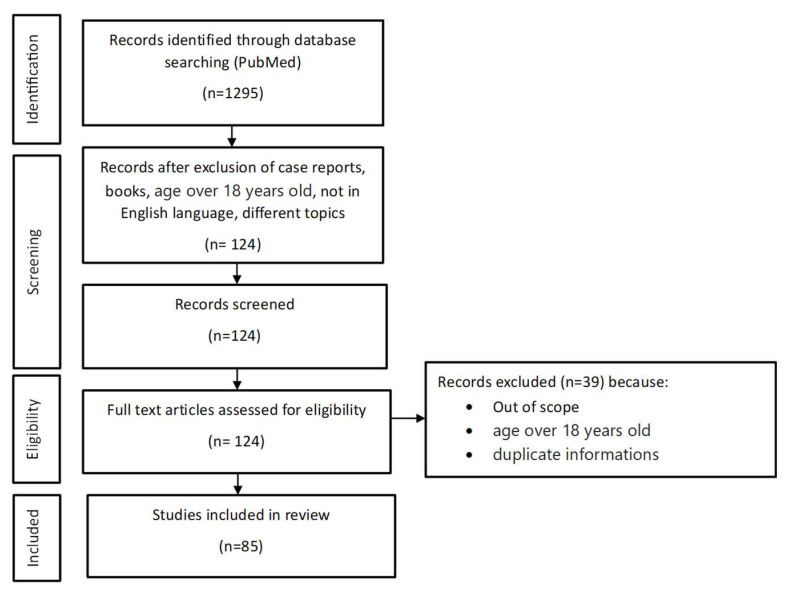
Flowchart of study selection and inclusion in the article.

A literature search of published data was conducted using the PubMed search engine. The authors reviewed the current literature from the years 2018–2023. The following search terms were used: “uveitis in children”, “uveitis in children etiology”, “uveitis in children treatment”, and “uveitis in children diagnosis”, with similar terms used in conjunction with each pathogenetic factor described in the subsequent review. Additionally, references cited in the identified articles were reviewed to find additional reports. The articles used were in English only and were reviewed by title and abstract. For the review, we included articles on uveitis in children (under 18 years of age). The focus was mainly on meta-analyses, reviews, and systematic reviews; clinical trials and articles on adult populations were included only if they provided new insights into the characteristics, diagnosis, or treatment of the disease entity. Below is a figure illustrating a simplified selection process. Figure 1.

## 3. Results

### 3.1. Etiology

The incidence of uveitis in the pediatric population is 4.3 per 100,000, and its prevalence is 27.9 per 100,000 [4]. Based on etiology, uveitis can be divided into three categories: infectious, non-infectious, and idiopathic. Of these, idiopathic is the most common, representing 40% of cases [5]. For identifiable etiologies, the non-infectious type of uveitis is predominant (67.2–93.8%) [2], while juvenile idiopathic arthritis (JIA) is the most commonly known disease entity causing uveitis in children (21%) [3]. The infectious type accounts for about 3.5–8% of childhood uveitis [6]. In general, uveitis occurs with the same frequency regardless of gender. It is most common in white and black non-Hispanic children [3].

The prevalence of different types of uveitis varies depending on geographical distribution, as shown in the table below. This table presents data from cohort studies in various countries conducted with minor patients, including the most common forms of uveitis in children. Table 1 [7,8,9,10,11,12,13,14,15,16,17,18].

The clinical presentation of uveitis can provide diagnostic clues to facilitate the identification of the relevant disease entity underpinning the ongoing inflammatory process in the eye. Table 2 [2,13,19,20] and Table 3 [21,22,23,24,25,26,27,28,29,30,31,32] list the symptoms characteristic of uveitis for each disease entity, categorized by infectious and non-infectious agents.

### 3.2. Diagnostic Methods of Uveitis Used in Children

#### 3.2.1. Infectious Etiology

The diagnosis of uveitis of infectious etiology is a difficult task, since the disease entities (infectious or non-infectious) that are the pathogenetic factor of uveitis may have an overlapping clinical picture [33]. Diagnosis is based on characteristic symptoms observed upon ophthalmologic examination, confirmed by laboratory tests. The laboratory tests used include the polymerase chain reaction (PCR) method, which has a higher efficiency than in vitro culture [34,35,36], serological methods, detection of anti-IgM and/or IgG antibodies, differential agglutination test (HS/AC test), latex agglutination and indirect agglutination tests (LAT test), immunosorbent agglutination test (ISAGA), immunochromatographic tests (ICT), enzyme-linked immunosorbent assay (ELISA), Sabin–Feldman stain test (DT), indirect fluorescence assay (IFA), direct agglutination test (DAT), IgG avidity test, and Western blot (WB) analysis [37].

A retrospective study from South Korea described the superiority of PCR over serological testing in diagnosing infectious uveitis; the percentage of initial diagnoses using PCR was 28%, while it was only 11% for serological testing. The utility of PCR testing has been demonstrated, especially for uveitis caused by cytomegalovirus, chickenpox, and hemiparetic virus, as well as Toxoplasma gondii [38]. A modern technology that has been used with increasing success in the diagnosis of IBD is metagenomic next-generation sequencing (mNGS), which has been used in the detection of herpes simplex virus (HSV) and cat scratch disease, among others [36,39].

#### 3.2.2. Non-Infectious Etiology

In the diagnosis of non-infectious factors responsible for the development of uveitis in children, multispecialist cooperation plays a key role, since the substrate of uveitis in this case is severe systemic diseases. The diagnosis is made based on the presence of systemic-specific criteria and characteristic ophthalmologic findings. Various imaging methods are used in ophthalmic diagnosis.

Slit lamp biomicroscopy is an essential test used in every age group and allows imaging of complications such as anterior and posterior adhesions, lens opacities, inflammatory cells, vitreous body opacity, band keratopathy, corneal deposits (KP), and inflammatory cells in the anterior chamber. This method requires the cooperation of the patient, which is particularly difficult in the case of pediatric patients.

Indocyanine angiography (ICGA) is a method involving the intravenous administration of indocyanine dye, which is essential for detecting subclinical choroidal inflammation and monitoring responses to treatment. It has been used successfully in the diagnosis of uveitis in the course of tuberculosis [34], Blau syndrome [40], systemic lupus erythematosus [41], and Vogt–Koyanagi–Harada disease [42].

Optical coherence tomography (OCT) is a method that is well tolerated by children, in contrast to the difficulty or lack of cooperation with the patient during slit lamp examination used in the diagnosis of CSF, especially of the posterior segment. This examination provides an objective assessment of the progression or regression of the disease and allows assessment of macular degeneration and edema, which can cause visual impairment in about 8% of children with uveitis [43]. Maculopathy can be detected by OCT in 84% of cases of uveitis associated with JIA [44]. Anterior segment OCT is a method that allows for the quantitative evaluation of cells in the anterior chamber, which requires refinement of standardization for routine use [45]. Its use in children has been described, for example, in Blau syndrome as a test to improve the diagnosis of retinal and corneal lesions in children with this condition [46,47].

Optical coherence tomography angiography (OCT-A) is a method for evaluating the vasculature and microcirculation in the retina and choroid of the eye. It allows for the visualization not only of the superficial choroid plexus (like FA) but of the entire retinal vascular system. It allows for detailed imaging of such pathologies as vascular obstruction and neovascularization [48]. A 2022 Chinese study showed that retinal and choroidal vascular density decreases, while retinal thickness significantly increases, in patients with autoimmune posterior segment uveitis [49].

Fluorescein fundus angiography (AF) allows for the assessment of uveitis activity by imaging complications such as cystoid macular edema (CME), retinal vascular exudate, choroidal and retinal lesions, lack of perfusion, and neovascularization. It is relatively difficult to perform in younger patients without pharmacological sedation, as imaging often involves pupil dilation and intravenous injection of fluorescein, followed by a series of fundus photographs. Nowadays, it is possible to administer the dye orally, which promotes the child’s psychological comfort during the examination [50]. There are reports that the sublingual administration of fluorescein requires the use of a lower dose, due to a reduced first-pass effect through the liver [51]. Pathologic changes depicted with AF may have prognostic value; in Behçet’s disease, such lesions as an ischemic macular area, exudative changes of the macular area, posterior and diffuse retinal vasculitis, lack of peripheral capillary perfusion, excessive retinal vascular leakage, hyperfluorescence of the optic nerve disc, neovascularization of the optic disc, cystic macular edema, and arteriolar stenosis have been associated with poor prognosis [52].

Fundus autofluorescence (FAF) provides information about the state of the retinal pigment epithelium (RPE). The fluorescence is derived from lipofuscin, which is the eye’s natural pigment and a byproduct of intracellular metabolism in the photoreceptors and RPE. Its measurement provides information about the metabolic state of the retina. This test is an essential diagnostic tool in Eales disease. The early venous phase illustrates the staining of inflammation-affected vessel walls. In the late phase, pigment extravasation takes place. Neovascularization in the arteriovenous phase is manifested by hyperfluorescence, and hardened capillaries may show a lack of perfusion-hypofluorescent areas [53].

Ultra-wide field (UWF) imaging in uveitis can be used to detect secondary complications or monitor disease activity. The examination does not require pupil dilation, so it is particularly useful for posterior adhesions. There are different types of confocal scanning laser ophthalmoscopes (cSLO). A commonly used one is from Optos, which images the retina over a previously unattainable angular range of 200 degrees [52]. Another UWF system, the Clarus 500, allows imaging of the retina in color at a higher resolution, which more consistently corresponds to the image observable in a fundus examination, but the recorded field does not exceed 133° [54]. At present, it is uncertain whether the Clarus 500 can be used for pediatric uveitis [48]. A study conducted in Los Angeles in 2019 showed that UWF does not require sedation during the examination in children [55] and is used in the diagnosis of a number of conditions manifested by uveitis, including Vogt–Koyanagi–Harada syndrome (VKH), where a system was developed to grade the characteristic fundus image. The sunset glow fundus (SGF) was based on pigmentary changes seen on UWF imaging as early, intermediate, and advanced forms (showing increased risk of cataracts, glaucoma, and chronic uveitis) [56]. In Behçet’s disease, UWF enables the monitoring of disease activity as well as treatment decisions. A 2017 London study described that as many as 43.4% of lesions detected with UWF could not be visualized with standard AF [52]. A 2016 Indian study demonstrated the utility of UWF in Eales disease. The test helped identify peripheral retinal lesions (in 67% of eyes), accurately localize a lack of vascular perfusion (in 54% of eyes), and determine vascular involvement (in 21% of eyes). In 33% of eyes, the previous treatment plan was changed due to the detection of lesions with UWF angiography [57]. The use of UWF has also been described in the infectious diseases cytomegalovirus infection [58] and tuberculosis [35].

Ultrabiomicroscopy (UBM) is an ultrasound examination of the anterior segment of the eyeball using a special probe, allowing for the evaluation of the ciliary body, pars plana, and areas of the vitreous body behind the iris. It is a useful tool for evaluating hypotony of the uveal membrane of the eye, and allows differentiation between hypotony against a background of mechanical damage to structures and that caused by chronic inflammation [59]. The use of UBM requires patient cooperation, which is a significant limitation among pediatric patients [47].

### 3.3. Treatment

#### 3.3.1. Infectious Uveitis

The treatment of infectious uveitis is closely linked to specific pathogenetic factors, leading the authors to focus on describing the treatment of the most commonly diagnosed disease entities in the pediatric population.

##### Toxoplasmosis

The parasitic disease caused by the protozoan *Toxoplasma gondii* is one of the most common causes of posterior segment ophthalmoplegia in children [2]. Treatment indications for ocular toxoplasmosis in children include optic nerve head involvement, involvement of the posterior uveal membrane by focal lesions located in the macula and between the vascular arcades, dense vitreous body opacities, lesions larger than three optic nerve disc diameters, and immunocompromised patients and neonates with congenital toxoplasmosis, regardless of clinical symptoms [60]. Treatment is based on antiparasitic therapy with pyrimethamine and sulfadiazine, used alongside steroid therapy. The literature also highlights the benefits of long-term cotrimoxazole therapy in children to reduce recurrence incidence [43]. Due to the risk of infection reactivation (as antiparasitic treatment may be ineffective in controlling cysts in chronic forms of the disease), children with toxoplasmic post-inflammatory lesions in the retina and choroid should be under continuous monitoring [43,61]. During antiparasitic treatment, patients may experience adverse drug reactions, which can include leukopenia and thrombocytopenia [62].

##### HSV

Targeted treatment for HSV-induced conditions includes oral antiviral drugs [60] such as acyclovir and valacyclovir, which have reported similar efficacy. Oral acyclovir is more potent than its topical form [63]. Topical glucocorticosteroids (GCSs) are also used to relieve inflammation. There are no reports of adverse effects of acyclovir occurring exclusively in pediatric patients. The most commonly reported complications from antiviral treatment with acyclovir in the general population include conjunctivitis, epitheliitis, uveitis, keratoconjunctivitis, corneal creeping ulcer, increased intraocular pressure, and swelling of the eyelids and face [64]. The most serious ocular complication of HSV infection in children is acute retinal necrosis [65]. Its characteristic clinical presentation includes rapidly progressive peripheral necrotizing retinitis, retinal and choroidal vasculitis, and vitreous body inflammation, facilitating preliminary diagnosis. Confirmation requires PCR testing and analysis of the intraocular fluid using the Goldmann–Witmer coefficient [43,60].

##### Tuberculosis

In the treatment of tuberculosis in pediatric patients, a standard regimen is recommended. For the first two months, either four or three drugs are used: rifampicin, isoniazid, pyrazinamide, and optionally ethambutol. This is followed by a two-drug regimen of isoniazid and rifampicin for another four months to eliminate the remaining mycobacteria. For severe forms of the disease—such as bone, disseminated, and cerebral tuberculosis, and extensive pleural and pulmonary disease—the treatment duration can extend up to 12 months [66]. The initiation of antituberculous therapy in cases of uveitis should be considered in the presence of any immunological evidence of tuberculosis. However, due to increasing drug resistance, treatment should not be introduced without some clinical evidence [63]. While there is no test that can confidently diagnose pediatric tuberculosis, the diagnosis should be based on the clinical course of the disease combined with imaging findings and immunological tests [67]. Antituberculosis treatment regimens in pediatric patients are generally safe and carry a low risk of side effects, according to the literature [67]. However, there are reports of a worsening condition in some children, resulting from a fulminant inflammatory reaction after the initiation of treatment [68]. In justified cases, classical antituberculous therapy can be supplemented with steroids or immunosuppressive drugs [63].

##### Cat Scratch Disease (CSD)

The disease caused by the bacterium *Bartonella henselae* typically follows a self-limiting course in individuals without immunodeficiency. Diagnosis is aided by a history of contact with cats and positive serological or PCR tests. While the literature lacks specific treatment regimens for the ocular manifestation of CSD [69], improvements in visual acuity after systemic antibiotic therapy have been documented [39]. In children, the recommended first-line antibiotics are azithromycin and rifampicin; if these are ineffective, the addition of gentamicin should be considered. The duration of therapy varies depending on the involvement of other organs and the clinical response to the treatment [70]. Monitoring therapy should include evaluating inflammatory parameters and possibly conducting imaging studies. There are reports supporting the benefits of combining systemic antibiotics with glucocorticosteroids (GCS) in patients with moderate to severe CSD, including children [39].

##### Borreliosis

Antibiotics with good intraocular penetration include cefazolin, cephalosporins, ceftriaxone, meropenem, and ceftazidime [71]. In the treatment of the ocular form of Lyme disease, the literature describes the use of orally administered moxifloxacin and linezolid, as well as trimethoprim and disulfiram for the long-term treatment of recurrent forms [72]. Topical treatments also involve intravitreal injections of vancomycin, ceftazidime, moxifloxacin, oxacillin, cefazolin, clindamycin, erythromycin, and ampicillin. This topical treatment can be supplemented with intravenous administration to prolong the antibiotic’s action [73].

#### 3.3.2. Noninfectious Uveitis

Treatment of IBD in most cases is based on causal therapy. The most commonly used drugs are corticosteroids (GCS) in monotherapy or in combination with biologic therapy, applied topically, periocularly, intralesionally, or systemically. The route of administration depends on the severity of the disease process, the presence of complications, and patient cooperation. First-line drugs are topical GCSs, most commonly 1% prednisolone acetate. While 0.05% difluprednate (Durezol) shows a more potent effect, its use is limited because it contributes to a clinically significant increase in intraocular pressure (IOP) [74]. Acetonide triamcinolone (TA), administered by periocular or intravitreal injection, allows the drug to penetrate deeper structures (intermediate segment IOP, posterior segment IOP, or panuveitis). A 2019 study among the adult population showed the superiority of intravitreal TA therapies over periocular injections in the treatment of uveitis macular edema [75]. Long-acting therapies include Ozurdex, a dexamethasone implant that is effective for 6 months [76], and Retisert, a fluocynolone acetonide implant that acts over the course of approximately 3 years [77]. They are used only as a last resort due to the increased risk of glaucoma and cataracts. Inflammatory conditions refractory to local treatment require systemic steroid therapy. Long-term (more than 3 months) GCS therapy is not recommended due to side effects: increased intraocular pressure, glaucoma, cataracts, and retinal and choroidal emboli, as well as growth and developmental retardation, gastrointestinal complaints, psychosis, and dermatological (hirsutism, striae, and impaired wound healing) and hormonal (adrenal inhibition, hypertension, hyperglycemia, and weight gain) complaints.

Therefore, the early implementation of therapy with disease-modifying (anti-rheumatic) drugs, sparing the administration of corticosteroids [43], which include methotrexate (MTX), mycophenolate mofetil (MMF), and cyclosporin A (CsA), is necessary in children with chronic uveitis. Methotrexate is a frequently used drug due to its good tolerability and efficacy in children. A 2013 meta-analysis found that about three-quarters of children with chronic uveitis treated with methotrexate can expect the regression of intraocular inflammation [78,79]. Subcutaneous injections of methotrexate are better tolerated and have higher bioavailability than the oral regimen. The achievement of its full therapeutic effect occurs after 3–4 months. Folic acid supplementation prevents toxic side effects. The discontinuation of MTX is associated with a high risk of relapse among most children within 2 years of gradual dose reduction or cessation of treatment [80]. Guidelines from the American College of Rheumatology/Arthritis Foundation and the Single Hub and Access Point for Children Rheumatology in Europe (SHARE) initiative recommend at least 2 years of remission without topical GCSs before attempting to phase down MTX [81]. If MTX is contraindicated, CsA or MMF can be used, with moderate efficacy.

Biologic drugs are used in refractory cases or to control inflammation in combination with MTX in children with severe uveitis at diagnosis. The most commonly used biologic therapies include tumor necrosis factor (TNF) inhibitors and monoclonal antibodies against interleukin 6 (IL-6) and B and T lymphocytes.

There are two anti-TNF drugs most commonly used in the treatment of refractory uveitis. The first is adalimumab, available for use from 2 years of age. It shows few allergic reactions and can be administered subcutaneously [82]. The standard dosing regimen is 20 mg in children weighing <30 kg and 40 mg in children weighing ≥30 kg, administered every other week. The Boston 2022 study also showed that weekly dosing was effective and safe in children not responding to the standard dose [83]. The second, infliximab, is a drug used as an alternative treatment for NSAIDs when there is no response to a combination of GCSs with adalimumab [84]. If anti-TNF drugs are not effective, alternative biologic drugs such as tocilizumab, abatacept, or rituximab can be used. Work is underway to determine the efficacy and safety of oral baricitinib, a selective inhibitor of Janus kinase (JAK) 1 and 2, in patients with active chronic uveitis associated with JIA and in ANA-positive patients in whom methotrexate therapy remains ineffective or is poorly tolerated [85].

## 4. Discussion

We conducted a literature review on uveitis in the pediatric population and presented the most recent data on its epidemiology, clinical manifestations, diagnostic methods, and treatment of the most common disease entities. Although uveitis in children is not a common condition, affecting only about 0.03% of the population, it can lead to serious complications and often represents the first manifestation of various systemic conditions. The literature indicates that the prevalence of disease entities underlying uveitis in children varies by geographic location. Childhood uveitis is most often idiopathic, with JIA being the most common identifiable cause. The authors described the most characteristic ocular manifestations, which can aid in making the correct diagnosis.

In diagnosing pediatric uveitis, it is crucial to take an appropriate history from either the patient or their caregivers and to consult with a pediatrician to comprehensively assess the child and their symptoms. For infectious uveitis, after considering exposure factors, clinical signs, and possible organ changes, a variety of laboratory tests are used depending on the suspected pathogen, with PCR often being preferred over in vitro cultures or serological tests. The modern method of mNGS is also gaining popularity. Pediatric non-infectious uveitis often indicates severe systemic diseases and might be their first manifestation, making ophthalmologic diagnosis particularly vital not only for improving visual acuity and preventing ocular complications but also for potentially identifying underlying systemic diseases. Imaging studies are essential in non-infectious uveitis. The literature notes that the major diagnostic challenge in pediatric uveitis is the limited ability of minors to cooperate during most examinations. OCT is highlighted as being well tolerated by children, providing definitive and objective results. AF imaging can be facilitated and made more comfortable for children by administering the dye orally, which also allows for a lower dose of fluorescein. The UWF method does not require sedation or pupil dilation and proves useful in diagnosing both infectious and non-infectious uveitis.

Treatment of the infectious forms of uveitis primarily focuses on pathogen elimination, typically through systemic therapy regimens. Although clear management regimens for pediatric uveitis, such as in cases of Lyme disease and CSD, have not yet been established, the literature often only describes standards for the adult population. In children, the knowledge mainly comes from individual clinical case descriptions. In addition to targeted therapy, systemic or topical GCSs are commonly used to reduce inflammation, often in combination with causal treatments.

In the treatment of non-infectious uveitis in children, therapy typically starts with glucocorticosteroids regardless of the etiology. If therapeutic effects are unsatisfactory, it is followed by disease-modifying drugs (MTX, MMF, and CsA). MTX is often chosen for children due to its good tolerability and efficacy, with a preference for subcutaneous injections. In severe, treatment-resistant cases, biologic therapy is employed, with adalimumab being the most preferred drug in pediatric patients.

To ensure optimal treatment outcomes for sick patients, multidisciplinary care is essential, regardless of the cause.

The major limitation of this review is the absence of clear guidelines in the literature, derived from multicenter studies on large pediatric cohorts, specifically for the treatment of uveitis. Additionally, considerable discretion exists in the choice of diagnostic methods and therapeutic options. Some of the data included in this paper are drawn from studies conducted on adults due to a lack of similar research involving minors.

## Figures and Tables

**Table 1:** Etiological factor of uveitis in children—depending on geographical distribution.

Country	Etiological Factor of Children Uveitis (Percentage of Children with Uveitis)										
	Toxoplasmosis	Herpes Viruses	CSD	Tuberculosis	JIA	Sarcoidosis	TINU	Behçet’s Disease	JSpA	SLE	VKHD
Poland [7]	21.80%	1.56%	-	4.69%	-	3.10%	-	-	1.50%	1.50%	-
India [8]	11%	-	-	3%	-	5.00%	-	2%	6.00%	-	5%
Italy [9]	4.45%	4%	-	0.63%	-	-	-	-	-	-	-
Great Britain [10]	1.6–15.2%	-	-	-	-	-	4%	-	15%	-	2%
Lebanon [11]	7.2–25%	2%	-	2%	12%	2%	2%	6.10%	-	0.80%	2%
France [12]	-	1.20%	-	-	25.60%	2.30%	1.20%	1.20%	3.40%	-	-
Greece [13]	-	-	15%	-	-	-	-	-	-	-	-
Canada [12]	-	-	18.50%	-	-	-	-	-	-	-	-
Chile [12]	-	-	13.30%	-	-	-	-	-	-	-	-
Finland [14]	-	-	-	-	61%	-	3%	-	-	-	-
Korea [15]	-	-	-	-	14.80%	-	1.30%	6.50%	-	0.70%	1.90%
China [16]	0.50%	-	-	-	8.10%	-	1%	1.90%	1.90%	-	3.30%
Jordan [17]	-	-	-	-	35.50%	-	1%	11.40%	3.10%	-	3.10%
Egypt [6,18]	-	-	-	-	6.70%	9.90%	-	9.90%	2.80%	-	3.30%

CSD—cat scratch disease; JIA—juvenile idiopathic arthritis; TINU—tubulointerstitial nephritis and uveitis; JSpA—juvenile seronegative spondyloarthropathies; SLE—systemic lupus erythematosus; VKHD—Vogt–Koyanagi–Harada disease.

**Table 2:** Infectious agents involved in the pathogenesis of pediatric uveitis—ocular manifestations.

Disease Entity	Characteristic Ocular Physical Symptoms
Toxoplasmosis	Focal necrotizing retinitis and choroiditis with focal unilateral vitreous inflammation and granulomatous anterior segment NSAID [2].
Herpes viruses	Unilateral uveitis and/or keratitis, inflammatory cells in the anterior chamber, atrophy of the iris, increase in intraocular pressure [19].
Cat scratch disease (CSD)	Parinaud’s eye-node syndrome involving the conjunctiva and regional lymph nodes account for about 5% of cases. Optic neuritis and starry macular figure (“neuroretinitis”) may also occur. Most common early symptom: unilateral anterior segment NBS [13].
Tuberculosis of the eye	Granulomatous lesions occupying the posterior segment or intermediate part of the uveal membrane. In millet form, small tubercles (white or yellow inflammatory foci) are visible. Complicated course: subretinal neovascularisation and inflammatory reaction in the vitreous body [20].

**Table 3:** Non-infectious factors involved in the pathogenesis of pediatric uveitis–ocular manifestations.

Disease Entity	Characteristic Ocular Physical Symptoms
JIA	The most common cause of anterior segment uveitis in children (50–80%). Chronic non-malignant uveitis, presence of inflammatory cells and proteinaceous deposits in the anterior chamber, and saddled corneal deposits. Ocular complications: band keratopathy, posterior adhesions, cataracts, glaucoma, hypotony, macular edema, epithelial membrane, and edema of the optic disc [21].
Sarcoidosis	Keratin precipitate (KP) visible on corneal endothelium/small granular KP, iris nodules, barrel reticular nodules, peripheral tent-shaped anterior adhesions, vitreous body opacities with visible snowballs/strings of pearls, multiple peripheral choroidal and retinal lesions, nodular/segmental perivascular inflammation (candlewax drippings), retinal macroaneurysm, optic nerve disc granulomas, or a single choroidal nodule, lesions usually bilateral [22].
Blau syndrome	Mostly bilateral uveitis. Edema, optic disc pallor, macular edema, periorbital nodules, subepithelial corneal deposits, and multifocal choroidal and retinal lesions. Common complications: band keratopathy, posterior adhesions, increased intraocular pressure, and cataracts [23].
Tubulointerstitial nephritis and uveitis (TINU)	Bilateral nonmelanomatous inflammation primarily of the anterior uvea that occurred less than 2 months before or 12 months after the onset of interstitial nephritis in the absence of other more likely causes to explain the eye or kidney pathology [24].
Behcet’s disease	Most often bilateral posterior CNS, less often anterior CNS with hypopyon. Other observable signs: inflammation of the iris, ciliary body, cornea, epiretinal inflammation, vitreous hemorrhage, cataract, glaucoma, retinal detachment, and aphthous oral ulcer [25].
Inflammatory bowel disease (IBD)	Most commonly anterior segment CNS. Retinal vascular obstruction, orbital inflammation, myositis, papillitis, corneal infiltration, scleral softening, perforated scleromalacia, and optic neuritis [26].
Juvenile seronegative spondyloarthropathies (JSpA)	Unilateral anterior segment CSF. Possible presence of deposits on the corneal endothelium, inflammatory cells in the aqueous fluid, exudate in the anterior chamber, and the formation of post-inflammatory adhesions in this location [27].
Systemic lupus erythematosus in children (SLEc)	Dry keratoconjunctivitis, iritis, and ciliary body inflammation, as well as retinal vascular lesions, optic neuritis, and occult vasculitis [28].
Multiple sclerosis (MS)	In 45–94% of intermediate segment uveitis, other parts of the choroidal membrane may also be affected. The inflammatory process can be complicated by iris nodules, iris–lens adhesions, vitreous body exudates, periphlebitis, cystoid macular edema, exudates, retinal ischemia, and central retinal vein occlusion, as well as glaucoma and cataracts [29].
Idiopathic inflammation in the lower part of the pars plana, peripheral choroidalthe pediatric population (pars planitis)	The presence of “globules and snowdrifts” in the vitreous body in the lower part of the pars plana, peripheral choroidal sheaths, cystic macular edema, and epiretinal membrane. Complications include clouding of the vitreous body, macular edema, papillitis or vasculitis, and cataracts [30].
Vogt-Koyanagi-Harada disease (VKHD)	Bilateral ESRD, usually of the anterior segment with iris nodules with edema of the optic disc, multiple exudative retinal detachments, and vitreous inflammation [31].
Eales disease	Recurrent, usually short-lived hemorrhages into the vitreous body and obliterative retinal periphlebitis. Possible complications: neovascular glaucoma, macular edema, neovascularization, membrane in the vitreous body, and retinal detachment [32].
